# Tracing metabolic flux in vivo: basic model structures of tracer methodology

**DOI:** 10.1038/s12276-022-00814-z

**Published:** 2022-09-08

**Authors:** Il-Young Kim, Sanghee Park, Yeongmin Kim, Hee-Joo Kim, Robert R. Wolfe

**Affiliations:** 1 Department of Molecular Medicine, Lee Gil Ya Cancer and Diabetes Institute Gachon University School of Medicine, Incheon, 21999 Republic of Korea; 2 Korea Mouse Metabolic Phenotyping Center, Lee Gil Ya Cancer and Diabetes Institute, Gachon University, Incheon, 21999 Republic of Korea; 3 Department of Health Sciences and Technology, GAIHST, Gachon University, Incheon, 21999 Republic of Korea; 4 Department of Geriatrics, the Center for Translational Research in Aging & Longevity, Donald W. Reynolds Institute on Aging, University of Arkansas for Medical Sciences, Little Rock, AR USA

**Keywords:** Blood flow, Translational research

## Abstract

Molecules in living organisms are in a constant state of turnover at varying rates, i.e., synthesis, breakdown, oxidation, and/or conversion to different compounds. Despite the dynamic nature of biomolecules, metabolic research has focused heavily on static, snapshot information such as the abundances of mRNA, protein, and metabolites and/or (in)activation of molecular signaling, often leading to erroneous conclusions regarding metabolic status. Over the past century, stable, non-radioactive isotope tracers have been widely used to provide critical information on the dynamics of specific biomolecules (metabolites and polymers including lipids, proteins, and DNA), in studies in vitro in cells as well as in vivo in both animals and humans. In this review, we discuss (1) the historical background of the use of stable isotope tracer methodology in metabolic research; (2) the importance of obtaining kinetic information for a better understanding of metabolism; and (3) the basic principles and model structures of stable isotope tracer methodology using ^13^C-, ^15^N-, or ^2^H-labeled tracers.

## Metabolism: Monitoring metabolic flux with stable isotope tracers

Tagging biomolecules with stable isotopes of specific atoms can reveal details of the molecular inter-conversions of metabolism. The masses of the tracer isotopes used are greater than those of the more common atomic forms. This allows their movement through different metabolic pathways to be detected using mass spectrometry and modeling. Il-Young Kim at Gachon University School of Medicine in South Korea and colleagues focus their review on the use of stable, non-radioactive isotope tracers, especially, of carbon, nitrogen, and hydrogen, to study metabolism in live humans and other animals. They cover the basic model structures of tracer methodology that serve as the fundamental basis for various tracer methods available and the most recent applications. Their procedure is especially useful for monitoring the rates of metabolic inter-conversions, which can reveal aspects of health and disease.

## Introduction

Biological compounds in living organisms were once thought to be static until the pioneering work by Rudolf Schoenheimer and his associates in the 1930s, who first developed and used a stable isotope tracer technique using ^15^N-labeled amino acids to investigate the in vivo kinetics of protein turnover^1^. They demonstrated that the body protein pool is in a dynamic state of constant turnover (i.e., protein synthesis and protein breakdown)^1^. In his posthumously compiled book in 1946 entitled “The Dynamic State of Body Constituents”, Schoenheimer eloquently described the dynamic state of living systems as follows: “all constituents of living matter are in a steady state of rapid flux”^2^. Indeed, virtually everything in the body turns over at varying rates to achieve overall “dynamic” homeostasis. A change in the pool size of any compound can occur when there is an imbalance between its rates of appearance and disappearance. For example, hyperglycemia (i.e., elevation of plasma glucose concentration above normal) can take place if the rate of appearance (*R*_*a*_) of glucose into circulation, typically through elevation of hepatic glucose output (sum of glycogenolysis and gluconeogenesis), exceeds the rate of disposal or disappearance (*R*_*d*_) of glucose from the circulation into various tissues for purposes such as glycogen synthesis, oxidation, and de novo synthesis of other compounds. Hyperglycemia can occur even when *R*_*d*_ glucose is elevated compared to the normal condition, provided that *R*_*a*_ glucose is accelerated to a greater extent. The relationships between the blood glucose concentration and the *R*_*a*_ and *R*_*d*_ of glucose underscore the importance of quantitative assessment of substrate kinetics in addition to measurement of the static concentration. Similarly, elucidation of the role of the activation state of molecular signaling molecules requires simultaneous quantification of the corresponding substrate kinetics. Snapshot measurements of molecular signaling pathways may not be consistent with the predicted substrate kinetics^3^. We will first discuss the importance of obtaining substrate kinetic data for a better understanding of the dynamic nature of substrate metabolism. We will then review the basic principles and models of stable isotope tracer methodology that can be used to assess in vivo kinetics at the whole-body and tissue levels.

## The dynamic nature of in vivo metabolism

**Fig. 1:**
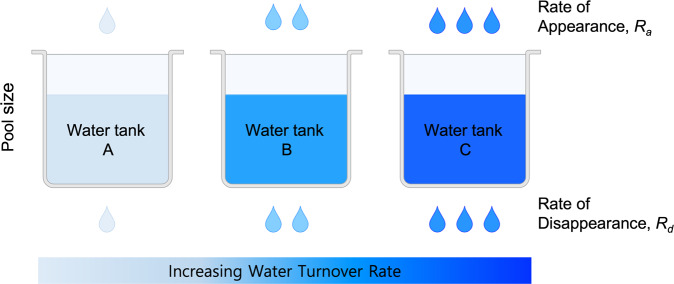
The volume of water (i.e., water pool size) in the tank is determined by the balance between the rates of appearance (*R*_*a*_) and disposal or disappearance (*R*_*d*_) of water. While the water pool size does not change if the two rates are identical, regardless of their absolute rates, differences in the water turnover rate may affect the quality of the water. The figure was created with BioRender.com.

The dynamic nature of the biological compounds in the body can be envisioned by considering the analogy of a water tank with flows of water in and out (i.e., water turnover). As shown in Fig. 1, it is intuitively clear that the volume of water in the tank is determined by the balance between the rate of appearance (*R*_*a*_) of “clean” water into the tank and the rate of disposal or disappearance (*R*_*d*_) of water from the tank. A steady state in the pool size achieved provided that the two rates are equal, regardless of the absolute rates (Fig. 1). However, the “quality” of the water in the tank may vary depending on the rate of water turnover despite the volume of water and other conditions being identical. To increase the volume of water in preparation for “drought”, *R*_*a*_ must increase and/or *R*_*d*_ must decrease. Indeed, there are an unlimited number of combinations of *R*_*a*_ and *R*_*d*_ that lead to the same volume of water (i.e., pool size) while achieving varying degrees of water “quality.” The amount of water in the tank (i.e., the pool size) is analogous to static concentrations and molecular activation states in biological systems and fails to provide critical kinetic information.

### Everything turns over in living organisms: synthesis of muscle proteins as an example

The balance between *R*_*a*_ (e.g., protein synthesis) and *R*_*d*_ (e.g., protein breakdown) determines the pool size of a specific individual protein (e.g., creatine kinase)^4–6^ in a subcellular organelle (such as mitochondrion)^7,8^, tissue/organ (e.g., skin, muscle, and liver)^9,10^, or even the whole body^11–14^. For example, in healthy adults, muscle mass is relatively constant because the loss of muscle proteins as a result of protein breakdown is replaced by continuous muscle protein synthesis. In growing children or resistance-trained adults who experience muscle hypertrophy, muscle mass increases when the rate of protein synthesis exceeds the rate of protein breakdown. On the other hand, in catabolic states such as sarcopenia or cancer cachexia, the opposite (i.e., breakdown > synthesis) occurs over time. Not only is protein pool size determined by the balance between the rates of synthesis and breakdown, identical pool sizes do not necessarily mean identical rates of protein turnover. Different rates of protein turnover can affect protein quality even when the protein pool size remains constant. For example, the quality of skeletal muscle, typically defined as muscle strength normalized to regional muscle mass^15^, is positively related to the rate of protein turnover^16^. Thus, it is important to maintain protein turnover (i.e., synthesis and breakdown) at or above a certain rate to maintain the optimal functional quality (the analogy of “clean water” in the water tank) of muscle by ensuring a constant supply of newly synthesized functional proteins (i.e., protein synthesis) to replace old, nonfunctional or damaged proteins that must be removed (i.e., protein breakdown). Dynamic homeostasis involves the continuous turnover of all body constituents at varying rates. Dysregulation of dynamic homeostasis will lead to physiological and pathological consequences.

### Static information does not reveal the dynamic nature of in vivo metabolism

Despite the dynamic nature of metabolism in vivo, metabolic research has historically relied on the analysis of static, snapshot information such as the abundance of metabolites, mRNA, and protein or the (in)activity of molecular signaling cascades^17^. Sole reliance on “static” information, collectively termed “statomics”, regarding the metabolic status of a living organism can result in misleading conclusions. A significant amount of evidence has documented mismatches between “statomics”, or the measurement of concentrations or molecular activation states, and metabolic dynamics in humans^3,18^ and animals^17,19^. For example, it was demonstrated that 48 h of fasting in rats resulted in significant elevation in the key enzyme for the first committed step in hepatic gluconeogenesis (GNG), i.e., phosphoenolpyruvate carboxykinase (PEPCK)^19^. Based solely on the expression of PEPCK, the conclusion would be that the 48-h fast increases GNG, whereas the corresponding in vivo flux rate (i.e., GNG) was actually reduced compared to control and 8-h fasting conditions^19^. This example of a mismatch between statomics and dynamic flux measurements is not limited to glucose metabolism^3,20^. Discrepancies between static information (e.g., enzyme abundance/molecular activation state) and actual metabolic flux rates for a given metabolic pathway occur because the actual fluxes of substrate metabolism are accomplished by complex interactions among various factors, including substrate availability, enzyme activity, and implicated signaling cascades^21^. In the following section, we will cover the fundamental principles of stable isotope tracer methodology and its use to assess in vivo metabolic flux rates, with a focus on the basic model structures of tracer methodology that apply to a wide range of applications.

## In vivo quantification of the dynamic nature of metabolism

### Basic principles of stable isotope tracer methodology

Information on in vivo substrate dynamics can be obtained with various tracer methods. Here, we will provide basic principles of tracer methodology with the emphasis on stable isotope tracers due not only to health issues related to the use of radioactive isotope tracers but also to the versatility of stable isotope tracers in assessing various aspects of metabolic status. Stable isotopically labeled tracers are any molecules with one or more heavier stable isotopes (e.g., ^13^C, ^2^H, or ^15^N isotopes) incorporated somewhere in the molecule^22–24^. Stable isotope tracers may be administered in the chemical form of the tracer (e.g., ^13^C glucose) or as heavy water (deuterium oxide, ^2^H_2_O) that will produce the desired metabolic tracer in vivo.

The calculation of substrate kinetics is predicated on two basic tracer models: (1) tracer dilution and (2) tracer incorporation^22,23^. These two basic models can be further divided into four tracer models depending upon the number of pools (single pool vs. multiple pools) and the number of precursors (single precursor vs. multiple precursors) in steady or non-steady states. These models may be used either independently or in combination to assess in vivo kinetics. We will discuss model structures in general terms because tracer methodology can be applied to a variety of different topics, and we will illustrate concepts with common examples. For simplicity, we will discuss these models in the postabsorptive state and physiological steady-state conditions. More comprehensive treatment of the topic can be found in the other references^22,23,25^.

## Tracer dilution model: single and multiple pools

Substrates turn over in living organisms in a single pool (e.g., fatty acids and water)^23,26–28^ or, more commonly, in multiple pools (e.g., glucose and amino acids)^23,29–31^. The tracer dilution model is based on the dilution of tracer administered into the system by the appearance in the same pool of unlabeled tracees. When an isotopic steady state is achieved, meaning that the rates of tracer and tracee appearance are constant over time and that there is a steady-state enrichment of the tracer in the body pools of the tracee, substrate kinetics can be calculated by the same method, regardless of the number of metabolic pools.

### Single-pool kinetics: free fatty acids (FFAs) as an example

We will discuss the example of palmitate, with the understanding that the principles of calculation apply to determining the kinetics of any other FFA and indeed any substrate distributed structurally or functionally in a single pool, such as water or plasma. In the case of palmitate, although there is exchange between the plasma and interstitial fluid, palmitate is considered to reside essentially in the plasma, as the exchange process between the two compartments (i.e., plasma and interstitial fluid) is too slow to be reflected in the kinetics of plasma palmitate^32^.

#### Model description

**Fig. 2:**
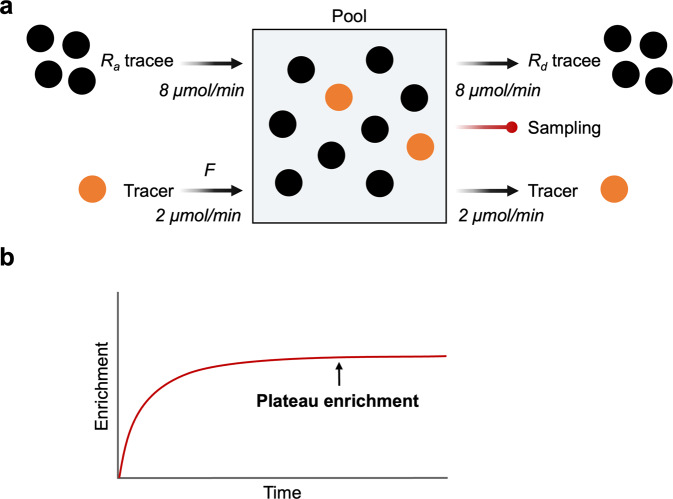
Tracer dilution model: single pool kinetics of a single substrate with palmitate as an example. **a** For a given tracer infusion rate of 2 µmol/min (*F*), the *R*_*a*_ of the tracee, a naturally occurring compound of interest to be traced, is equal to the *R*_*d*_ of the tracee in steady-state conditions where the pool size is constant and is determined as *F* divided by isotopic enrichment at plateau. **b** With time, tracer enrichment gradually increases and reaches isotopic equilibrium or plateau enrichment (*E*_*p*_), where the ratio of *R*_*a*_ tracee to F is equal to the ratio of *R*_*d*_ tracee to the rate at which tracer leaves the pool. After rearranging the relations, *R*_*a*_ tracee can be calculated as *F* divided by *E*_*p*_ during a continuous tracer infusion study. There is a linear correlation between the magnitude of tracer dilution (reduction in TTR) and *R*_*a*_ tracee for a given *F*.

The basic model structure for single-pool kinetics is depicted in Fig. 2. In a physiological steady state, the tracee pool size (i.e., concentration × volume of distribution) is constant over time, meaning that *R*_*a*_ tracee is equal to *R*_*d*_ tracee. *R*_*a*_ tracee and thus *R*_*d*_ tracee can be determined with either a bolus or continuous infusion of tracer. In the case of tracer infusion, the rate of infusion into plasma (*F*) is constant. In the initial time after the start of tracer infusion, tracer enrichment (i.e., tracer to tracee ratio, TTR) is relatively small but continuously increases over time in a single-exponential manner until reaching an isotopic equilibrium or plateau (Ep) where both the *F* and the “*R*_*d*_” of the tracer are constant and equal. For a given *F*, TTR is inversely related to the rate at which the naturally occurring unlabeled tracee appears in the plasma and dilutes the infused tracer.

#### Kinetic calculations

In the isotopic steady state, when a plateau in isotopic enrichment has been reached in the sampled pool, the ratio of *R*_*a*_ tracee to *F* will be directly reflected by the TTR. In Fig. 2, the ratio of the number of tracers (red circle, *n* = 2) and the number of tracees (black circle, *n* = 8), i.e., TTR, is 0.25 (2/8), which is dictated by the ratio of *R*_*a*_ tracer (i.e., *F*, 2 µmol/min) to *R*_*a*_ tracee (8 µmol/min). In the experimental setting, *R*_*a*_ tracee is unknown of interest, *F* is known, and TTR is measured. Because the ratio of *F* to *R*_*a*_ tracee dictates the ratio of tracer to tracee (i.e., TTR) at isotopic equilibrium, the following relation will be established: *F*/*R*_*a*_ tracee = Tracer/Tracee. By rearranging the relation, *R*_*a*_ tracee can be calculated as *R*_*a*_ tracee = *F*/TTR. *R*_*a*_ tracee in a single pool can also be determined with a bolus tracer injection. Isotopic enrichment in a single pool follows a single-exponential decay after a bolus injection of tracer, which enables the calculation of enrichment at time *t* over time using a single exponential equation (*E* = *E*_0_•e^−*kt*^); thus, *R*_*a*_ will be calculated as the amount of the bolus of tracer divided by the area under the curve of the decay curve of the enrichment.

## Other applications of single-pool model: D_3_-creatine dilution method assessing direct muscle mass

**Fig. 3:**
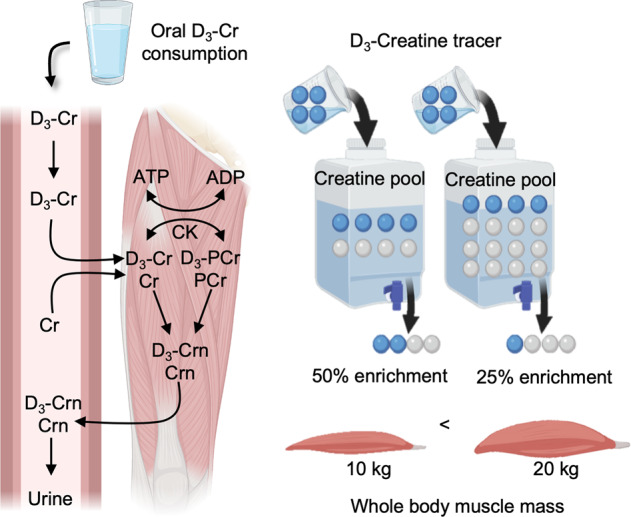
Basic principle of the D_3_-creatine dilution method assessing functional muscle mass. The method is predicated on the principle of tracer dilution: the magnitude of dilution of a given small dose of oral D_3_-creatine intake relative to the amount of preexisting creatine located predominantly in skeletal muscle, determined from the ratio of labeled creatinine to unlabeled creatinine in a urine sample, reflects total muscle mass. Labeled and unlabeled creatine are irreversibly converted to labeled and unlabeled creatinine in proportion to their relative concentrations and then excreted in urine. For example, the urine creatinine enrichment (i.e., ratio of D_3_-creatinine to creatinine) resulting from 20 kg of muscle will be half that resulting from 10 kg of muscle.

The single-pool model of tracer dilution is also applicable to other assessments, such as the direct determination of functional muscle mass, called the D_3_-creatine dilution method (Fig. 3). Briefly, the dilution of a small dose of oral D_3_-creatine is a direct function of the size of the endogenous creatine pool, which is primarily distributed in muscle. The ingested D_3_-creatine is distributed throughout the skeletal muscle pool of creatine, and subsequently both labeled and unlabeled creatine are irreversibly converted to creatinine in proportion to the relative abundances of labeled and unlabeled creatine. Measuring the creatinine pool enrichment as a reflection of the muscle creatine enrichment is advantageous because creatinine is excreted into the urine, meaning that the method is minimally invasive^33,34^. Therefore, the total creatine pool size can be calculated by dividing the dose of D_3_-creatine by the urinary enrichment of D_3_-creatinine at equilibrium^35^. Since the creatine pool size in skeletal muscle is relatively constant^36^, muscle mass can be calculated by dividing the creatine pool size by the muscle creatine concentration.

### Multiple-pool kinetics

Multiple-pool models apply to many substrates, including glucose and amino acids. We will discuss the multiple-pool model of a single substrate using glucose as the example.

#### Model description and kinetic calculations

**Fig. 4:**
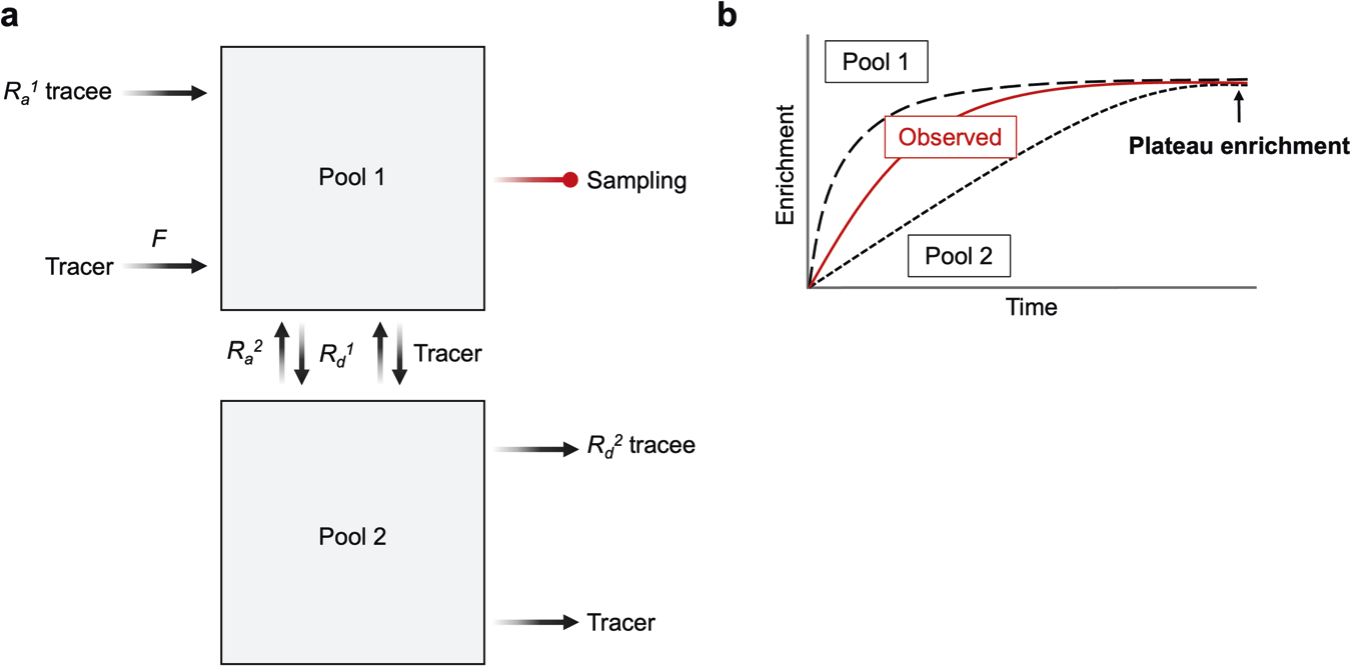
Tracer dilution model: multiple-pool kinetics of a single substrate with glucose *R*_*a*_ as an example. Substrates such as glucose reside in multiple pools. **a** For example, glucose dwells in plasma (pool 1) and interstitial fluid (pool 2), between which gradual glucose exchange occurs. In the postabsorptive state, glucose appears in pool 1 via endogenous glucose production and diffuses into pool 2, from which it is taken up by tissues. To determine *R*_*a*_ glucose (and thus *R*_*d*_ glucose) in steady state conditions, a tracer glucose is (typically) continuously infused into pool 1 at a specific rate (*F*). **b** The observed enrichment at the point of sampling (pool 1) reflects a mixture of the two pools. Regardless of the differences in the speed of mixing in the two compartments, an isotopic equilibrium will be reached where the isotopic enrichment levels of the two pools are identical. Under this condition, *R*_*a*_ glucose can be calculated as *R*_*a*_ tracee = *F*/*E*_*p*_. *R*_*a*_, rate of appearance; *R*_*d*_, rate of disappearance*, E*_*p*_, plateau enrichment.

Glucose resides in multiple pools (i.e., blood and interstitial fluid) and is one of the most commonly traced substrates. To simplify the conceptualization of the theory of the model structure, we will discuss the situation of a physiological steady state, meaning that *R*_*a*_ and *R*_*d*_ are equal and the blood level of glucose is constant over time. The basic principles of glucose kinetics are depicted in Fig. 4. Glucose enters the blood (pool 1) directly from the liver but disappears irreversibly into many tissues from the interstitial fluid (pool 2). There is constant exchange of both tracer and tracee glucose between pool 1 and pool 2. Although labeled and unlabeled glucose leave pool 2 to enter tissue, it is typically possible to sample only pool 1 (blood). Regardless, it is possible to use the same steady-state equations (i.e., *R*_*a*_ tracee = *F*/*E*_*p*_) in this model as for single pool kinetics, as isotopic exchange between pool 1 and pool 2 will eventually result in equal enrichment in the two pools (Fig. 4). In the case of glucose, isotopic equilibrium would be achieved much more quickly if it was distributed only in a single pool, but mixing in interstitial fluid is much slower than mixing in blood. Consequently, the observed increase in enrichment in blood (pool 1) occurs at a rate that reflects the time course of enrichment of both pool 1 and pool 2. The important point of Fig. 4 with regard to the calculation of steady-state kinetics is that since all the glucose that appears in pool 2 is derived from pool 1, the glucose isotopic enrichment in pool 2 will eventually reach the same equilibrium value as that in pool 1 (Fig. 4). Therefore, *R*_*a*_ tracee is calculated as *F* divided by *E*_*p*_, where *E*_*p*_ is the plateau enrichment measured in pool 1.

#### Bolus injection

In addition to the *R*_*a*_ of a single substrate distributed in multiple pools being determined with a constant tracer infusion, a bolus of tracer can be used. In the case of multiple-pool models, the measured decay curve in blood following tracer injection will be described by a multiexponential function. The number of exponential curves that best fit the decay data in a multiple model is related to, but not necessarily equal to, the number of physical pools. For example, the decay in glucose enrichment after a bolus tracer injection may be described by either a two- or three-exponential curve, even though the system behaves physiologically as a two-pool system^37^. Despite a multiexponential decay curve following a bolus tracer injection in a multiple pool model of a single substrate, *R*_*a*_ can still be accurately calculated from the area under the decay curve in a manner analogous to calculating *R*_*a*_ from the area under the decay curve in a single-pool system. Furthermore, by using the compartmental modeling approach^22,25^, additional kinetic parameters, including individual pool sizes, decay constant rates (*k*), half-life, and mean residence time, can be calculated.

#### Non-steady-state kinetics: Steele equation

The calculation of *R*_*a*_ of a single substrate distributed in multiple pools becomes much more complicated in a physiologically non-steady state. The calculation of *R*_*a*_ glucose in the non-steady state using a constant tracer infusion has been the most widely examined approach. The fundamental problem arises from different rates of mixing of the tracer and tracee in the different pools. Thus, with a rapid change in *R*_*a*_ in conditions such as the start of exercise^38–42^ or food intake containing carbohydrates^43,44^, the changing *R*_*a*_ glucose mixes rapidly with the tracer in pool 1 (i.e., blood), from which blood samples are taken and enrichment is measured for calculations of glucose kinetics. As a result, the blood enrichment in the early response of non-steady state conditions largely reflects pool 1 rather than the entire distribution volume of glucose. The mixing between pool 1 and pool 2 occurs more slowly than mixing throughout pool 1. The more extensive the mixing between pools 1 and 2 is, the more closely the sampled compartment will reflect the entire glucose pool. The most commonly used solution to the problem of the sampled compartment reflecting pool 1 and an unknown portion of pool 2 goes back to the 1950s, when Steele proposed calculating *R*_*a*_ glucose in a non-steady state by assuming that the observed values of glucose concentration and TTR reflected pool 1 plus a fixed fractional contribution from pool 2^45^. The limitation of this approach is that the extent of mixing between pools 1 and 2 is time-dependent and not well represented by a single fractional value, particularly when the change in the magnitude and direction of change in *R*_*a*_ are complex. Regardless of the many published criticisms of the Steele equation for calculating non-steady-state kinetics, it remains the most popular approach in the absence of a better method. Although quantification of changes in *R*_*a*_ in the non-steady state can be problematic, it should be recognized that a basic characteristic of the model dictates that a decrease in enrichment during a constant tracer infusion can occur only because of an increase in *R*_*a*_, and vice versa. The interpretation of changes in isotopic enrichment in a non-steady state is thus always qualitatively valid.

### Arterial-venous (A-V) difference tracer dilution method

The traditional A-V difference method involves measurement of the difference between the arterial inflow of a substrate (i.e., arterial concentration × blood flow) to an individual organ or tissue (such as skeletal muscle) and venous outflow (i.e., concentration of venous blood draining the tissue of interest × blood flow). This method has been pivotal in determining the interorgan net flux of compounds^46^ but provides no information about the underlying mechanisms responsible for various responses of net substrate uptake or release. The inclusion of isotopically labeled tracers can greatly expand the metabolic insight provided by the A-V model. We will use the example of the measurement of muscle tissue synthesis and breakdown using a phenylalanine (Phe) tracer. We will describe a simple one-pool model, with the understanding that the addition of more complex model designs is possible^47^.

**Fig. 5:**
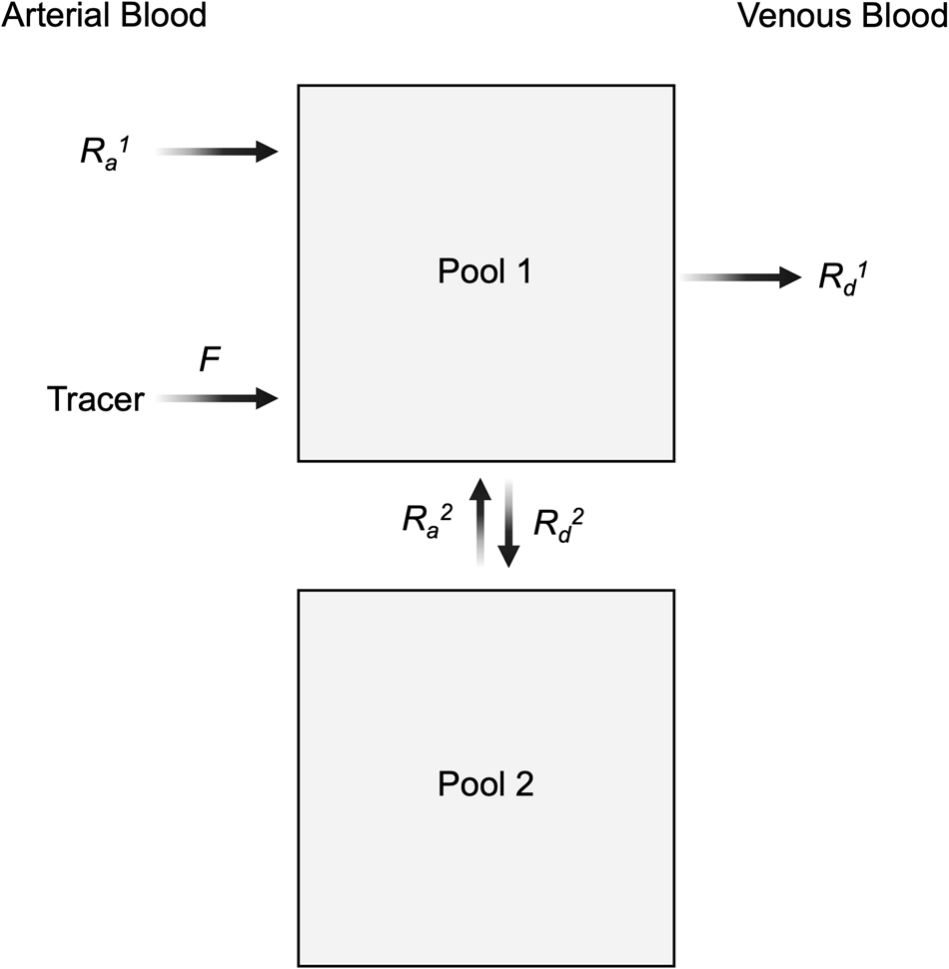
Arterial-venous (A-V) difference tracer dilution method with phenylalanine (Phe) kinetics across muscle as an example. The A-V model utilizes tracer dilution principles adapted for the measurement of tracer flux across individual tissues and organs. The example shown is for the measurement of muscle protein synthesis and breakdown using Phe as a tracer. For the purpose of explanation, the intracellular free Phe is represented as pool 1 and the protein as pool 2. More complex models, such as distinguishing the interstitial fluid pool from the intracellular free pool and distinguishing leg skin metabolism and leg muscle metabolism, are based on the same basic tracer dilution principles. Since Phe is an essential amino acid that cannot be produced in the body, the appearance of Phe in the intracellular pool of free Phe can come only from the blood (*R*_*a*_^1^) and from protein breakdown (*R*_*a*_^2^). *R*_*a*_^2^ is the difference between the total Ra (*R*_*a*T_) and *R*_*a*_^1^. *R*_*a*T_ is determined by the classic tracer dilution method, whereby the tracer *R*_*a*_ in pool 1 (blood flow × arterial Phe concentration × arterial Phe enrichment) is divided by the measured tracer/tracee ratio of Phe in pool 1. Since *R*_*a*_^1^ is measured (blood flow × arterial concentration of Phe), *R*_*a*_^2^ can be calculated. Once *R*_*a*_^2^ is known, the rate of protein synthesis (reflected as *R*_*d*_^*2*^) can be calculated as the sum of *R*_*a*_^1^ and the difference between *R*_*a*_^2^ and *R*_*d*_^1^. *R*_*a*_^1^ minus *R*_*d*_^2^ is the A-V difference of Phe.

The A-V tracer model relies on the same principles of tracer dilution models described above. The significant difference is the necessity of accounting for the blood flow into and out of the tissue. A schematic of the model is shown in Fig. 5. Since Phe is an essential amino acid that cannot be produced in the body, the appearance of Phe in the intracellular pool of free Phe (pool 1 in Fig. 5) can come only from the blood (*R*_*a*_^1^) and from the breakdown of protein (pool 1 in Fig. 5), reflected as *R*_*a*_^2^. *R*_*a*_^2^ is the difference between the total *R*_*a*_ (*R*_*a*T_) and *R*_*a*_^1^. *R*_*a*T_ is determined by classic tracer dilution, whereby *R*_*a*_ tracer into pool 1 (blood flow × arterial Phe concentration × arterial Phe enrichment) is divided by the measured tracer/tracee ratio in pool 1. It is optimal to measure the intracellular enrichment directly, but if it is not possible to obtain a muscle sample, it can be assumed that the tracer/tracee ratio in the venous outflow reflects the enrichment of free Phe in the muscle pool. Since *R*_*a*_^1^ is measured (blood flow × arterial concentration of Phe), *R*_*a*_^2^ can be calculated. *R*_*a*_^2^ is a direct reflection of protein breakdown since Phe cannot be produced by any metabolic pathway. Once *R*_*a*_^2^ is known, the rate of protein synthesis, denoted as *R*_*d*_^2^, can be calculated as the sum of *R*_*a*_^1^ and the difference between *R*_*a*_^2^ and *R*_*d*_^1^. In the case of a net synthesis of muscle protein, *R*_*a*_^1^ will be greater than *R*_*d*_^1^_,_ and the reverse will be true when *R*_*d*_^1^ is greater than *R*_*a*_^1^. *R*_*a*_^1^ minus *R*_*d*_^2^ is the A-V difference of Phe.

## Tracer incorporation model

The tracer incorporation model calculates the rate of synthesis of a product from the rate of tracer incorporation. There is a distinction between the calculation of the rate of production when the product has only one precursor and when there are multiple potential precursors. Determining the fractional synthetic rate (*FSR*) of protein from the rate of incorporation of an amino acid tracer precursor is a commonly used example of a single-precursor model. *FSR* is considered to be a single-precursor model because there are no other precursors that might dilute the increase in enrichment over time resulting from the incorporation of the tracer (amino acid) precursor. In contrast, calculating the rate of glucose production from a single tracer precursor, such as lactate, is an example of a multiple-precursor model. In a multiple-precursor model, the rate of increase in tracer enrichment in the product is a function not only of the rate of incorporation of the tracee into the product but also of the rate of incorporation of other (unlabeled) precursors. In the example of glucose, the rate of increase in the enrichment of glucose from lactate will be diluted to a variable extent by the simultaneous production of glucose from other precursors that are not labeled, such as glycerol.

### Single-precursor model: muscle protein synthesis as an example

The calculation of muscle protein *FSR* will be used as an example. The *FSR* represents the fraction or percentage of the total protein pool size that has been newly synthesized during a given period of time (%/time). The *FSR* of any form of polymer with repeating (mono or multiple) precursors, such as DNA^48,49^, RNA^50^, glucose (i.e., gluconeogenesis)^51^, fatty acids (i.e., de novo lipogenesis, DNL)^52^, and triglycerides^52,53^, can be determined. The calculation of *FSR* is predicated upon the precursor-product relation^54^. In muscle protein synthesis, amino acids are precursors, and muscle protein is the product. An individual amino acid tracer is used to calculate the *FSR* of muscle protein derived from that precursor. The total muscle protein *FSR* is then calculated by taking into account the percentage of the protein comprising the amino acid being traced. As *FSR* is a “fractional” term, calculation of the total synthetic rate requires the multiplication of *FSR* by the total pool size of the product. *FSRs* between groups or conditions can be directly compared if the protein pool size remains essentially constant throughout the experimental time frame or if there are no significant differences in protein pool sizes between groups. For example, *FSR* is a direct reflection of the response of muscle protein synthesis when a crossover design is used in which the responses to two different nutritional supplements are compared in the same subjects. In contrast, muscle protein *FSR* must be multiplied by muscle mass to make a reasonable comparison between a group of elderly women with depleted muscle mass and a group of young healthy males with significantly greater muscle protein mass.

#### Model description

**Fig. 6:**
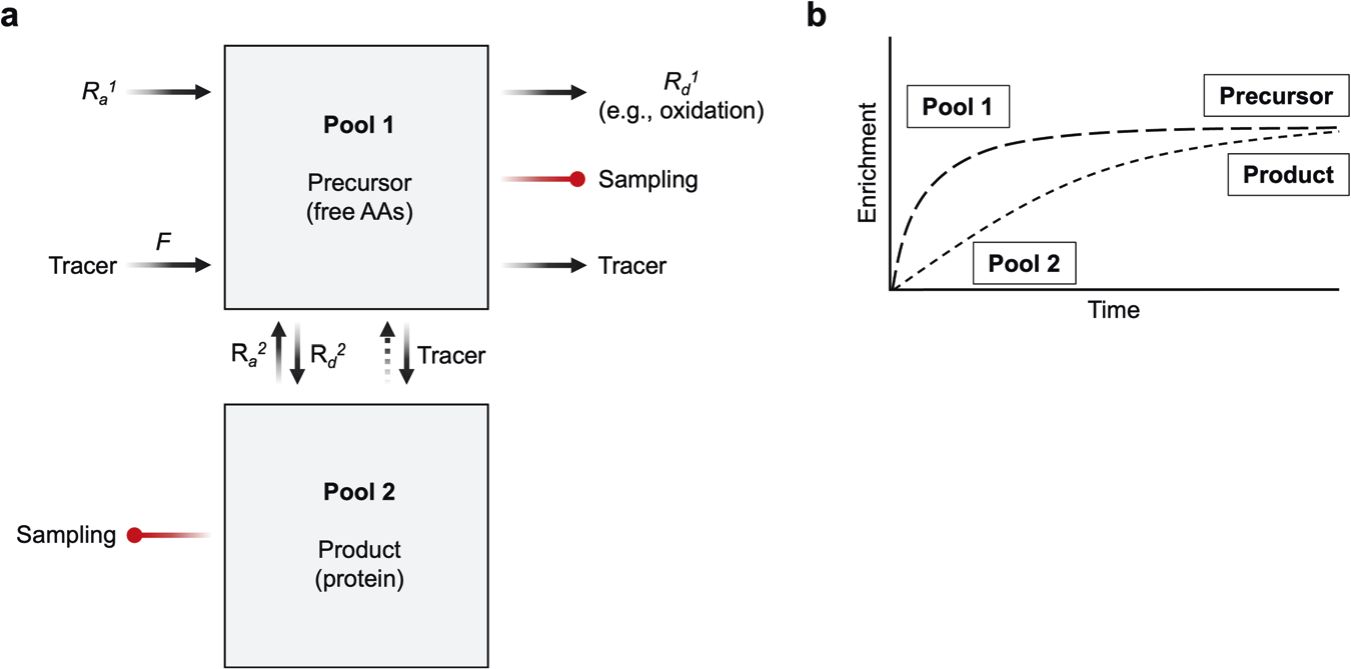
Incorporation model: single precursor and single product with the synthesis of new proteins as an example. **a** Both the labeled tracer (*F*) and the unlabeled amino acid tracee (*R*_*a*_^1^) enter pool 1 (intracellular precursor pool) in proportion to their relative rates of appearance, from which both tracer and tracee are either oxidized (*R*_*d*_^1^) or incorporated into protein (*R*_*d*_^2^). When using phenylalanine (Phe) as a tracer, *R*_*d*_^2^ Phe directly reflects the rate of protein synthesis, as irreversible loss of Phe by oxidation (more correctly, hydroxylation) does not occur in skeletal muscle. *R*_*d*_^2^ is the rate of muscle protein synthesis (*MPS*), calculated as the product of *FSR* multiplied by pool size. **b** *FSR* is defined as the % change in the product pool size resulting from new synthesis during a specified time interval. For example, for a continuous tracer infusion of essential amino acid, the calculation of *FSR* is based on the change in product enrichment between two time points (thus, two muscle samples obtained at *t*_1_ and *t*_2_), typically 2–8 h apart, divided by the precursor enrichment. The dotted line indicates that the rate of increase in product muscle protein enrichment is slow, and thus, during the experimental period, the product enrichment never approaches the precursor enrichment. Typically, the enrichment of both precursor and product are measured in the early phase of product enrichment but after precursor enrichment is plateaued. *R*_*a*_, rate of appearance; *R*_*d*_, rate of disappearance; *F*, infusion rate.

The basic principle of determining muscle protein *FSR* is illustrated schematically in Fig. 6. A stable isotope amino acid tracer is typically administered as a constant infusion or as a bolus injection (*F* in Fig. 6). Alternatively, labeled amino acid precursors can be produced in vivo from orally administered deuterium oxide, ^2^H_2_O. Tracer enrichment is diluted by the endogenous rate of appearance of the unlabeled counterpart of the tracer (i.e., tracee). If an essential amino acid is used as the tracer, the appearance of tracee in the fasted state is due entirely to release of that amino acid by protein breakdown (*R*_*a*_^2^ in Fig. 6), since essential amino acids cannot be synthesized in vivo. A nonessential amino acid can also be used as a tracer, but the appearance of tracees from protein breakdown and de novo synthesis must also be considered (*R*_*a*_^1^ in Fig. 6). The tracee and tracer will leave pool 1 (blood) for protein synthesis (*R*_*d*_^2^) or intracellular pathways other than protein synthesis, including oxidation (*R*_*d*_^1^). Tracer and tracee amino acids will leave pool 1 in proportion to their relative abundances in pool 1. There is an underlying assumption with the measurement of protein *FSR* that the turnover rate of the protein relative to its pool size is so slow relative to the experimental period that there is essentially no tracer recycling (i.e., appearance of tracer coming back from protein breakdown into the precursor pool)^47,55^. This assumption is valid in slow-turnover proteins such as muscle proteins^27^ but not in fast-turnover proteins such as apolipoprotein B (Apo B)^56^. In the case of Apo B, both the infused and recycled labeled amino acid precursor incorporate very rapidly into Apo B protein, and the product enrichment will rise quickly to a plateau, which represents the true precursor enrichment.

#### Kinetic calculations

An amino acid tracer (e.g., L-[ring-^2^H_5_]phenylalanine) is typically infused intravenously, and muscle protein is sampled (usually by needle biopsy) at the beginning and the end of tracer infusion. Protein *FSR* is calculated from the change in enrichment in the protein-bound tracer over a measured interval of time, divided by the enrichment of the precursor amino acid enrichment in the intracellular compartment: *FSR* (%/time) = [(E_BP2_ – E_BP1_)/(E_PC_ × *t*)] × 60 × 100, where E_BP1_ and E_BP2_ are the enrichments of bound tracer amino acid (e.g., L-[ring-^2^H_5_]phenylalanine) in the first and second biopsies, respectively, and E_PC_ is the precursor tracer enrichment, which is typically measured in the intracellular fluid of the muscle tissue sample. The time elapsed between the first and second muscle biopsies is represented by *t*. The factor 60 converts the units from minutes to hours, and the factor 100 expresses the result as a percentage rather than a fraction.

### Measuring “true” precursor enrichment

The correct, or “true”, precursor enrichment is key to the accurate calculation of *FSR*. The importance of precursor enrichment can be appreciated by considering two identical physiological circumstances, but in one case, the tracer infusion rate is twice that in the other. Tracer incorporation would be twice as fast with the higher rate of tracer infusion, even though the actual *FSR*s are identical. In the case of muscle *FSR*, it is possible to determine a reasonably accurate value for precursor enrichment by measuring TTR in the intracellular fluid of the muscle sample^57^. The intracellular free amino acid pool represents tracer precursor enrichment for cultured fibroblasts and myocytes^57^. In other circumstances, it may be more difficult to identify a reasonable surrogate for the true precursor enrichment. For example, intrahepatic acetate is the precursor for the de novo *FSR* of fatty acids, and that compartment is not available for sampling. Two different potential solutions to this issue were developed: mass isotopomer distribution analysis (MIDA)^58^ and isotopic spectral analysis (ISA)^59^. The basic principle is that true precursor enrichment can be back-calculated from the labeling patterns of the product^58,59^. A detailed description of these approaches can be found in the references^26,60,61^.

### Using deuterated water to assess the polymer synthesis rate in vivo

In addition to administered preformed tracer precursors, such as labeled amino acids, enriched precursors can be produced in vivo from “heavy water” (^2^H_2_O, deuterium oxide). The heavy water labeling of precursors has several potential advantages^62,63^ over the substrate-labeled tracer infusion method^11–14^. Most notably, heavy water labels a number of different precursors, which enables the simultaneous determination of *FSR*s of a variety of polymers with repeating (mono or multiple) precursor molecules, such as DNA^64^, fatty acids (de novo lipogenesis)^65^, and glucose (i.e., gluconeogenesis)^66^ in free-living conditions. Water moves rapidly and freely throughout the body, including across cell membranes, and equilibrates with all of the body fluid within ~30 min after administration^67,68^. However, there are several limitations of the heavy water method^24^. The time required for the measurement of *FSR* may be much longer than when a prelabeled precursor tracer is infused intravenously. For example, muscle protein *FSR* can be measured before and after a single meal when an amino acid tracer is infused intravenously at a constant rate, whereas the time frame of labeling an amino acid precursor from heavy water is too long for that experimental design to be practical^11–14,24,67,69^. In addition, there is no method to assess the fractional protein breakdown rate (*FBR)* using heavy water, although it is possible to infer changes in *FBR* over time from the measured *FSR* and changes in the protein pool size^70^. Finally, the amount of ^2^H_2_O that can be administered to a human without an adverse response may produce insufficient precursor labeling to easily detect its rate of incorporation into a slow-turnover compound.

#### Model description

**Fig. 7:**
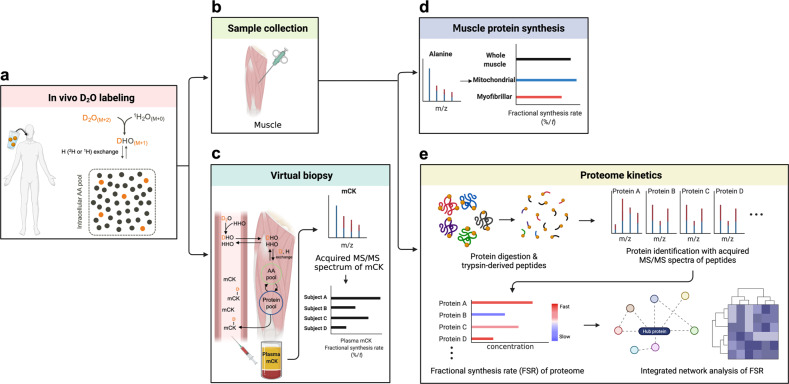
Heavy water labeling method for determining *FSR*s of various polymers. **a** A small dose of heavy water (deuterium oxide, ^2^H_2_O) is administered to achieve target enrichment (~1% for humans and ~5% for animals) with which to assess the fractional synthesis rate (*FSR*) of polymers such as muscle protein. ^2^H_2_O administered into the system will equilibrate with body water within a relatively short time (<30 min), forming monodeuterated water (i.e., ^2^H^1^HO). The ^2^H will exchange with H attached to various precursors, including amino acids. The number of exchanges and sites of exchange are known for a variety of compounds, including amino acids. For example, there are 4 sites in the case of alanine. The precursors labeled by exchange reactions with heavy water are incorporated into the products of interest, such as muscle protein. **b** Muscle protein *FSR* is typically determined from a muscle sample obtained by needle biopsy. **d** Specific compartments of a muscle sample can be labeled, such as contractile proteins, mitochondrial proteins, sarcoplasmic proteins, and pooled proteins, as well as the free intracellular precursor (e.g., alanine). **c** Muscle protein *FSR* can also be assessed without obtaining muscle tissues using the “virtual biopsy” technique. The virtual biopsy technique involves the measurement of tracer incorporation into specific proteins in blood that primarily reflect muscle protein metabolism. **e** In addition, *FSR* can be assessed at the proteome level, reflecting the *FSR* of several hundred or even thousands of individual proteins. The single-precursor incorporation model applies to the calculation of FSR in all the applications described in Fig. 7.

The basic principle of heavy water labeling to measure muscle protein *FSR* is depicted in Fig. 7a, b, d. Heavy water labeling is an approach to precursor labeling, and the determination of *FSR* relies on the same principles discussed above. To determine muscle protein *FSR*, ^2^H_2_O is administered (after bolus priming) orally. Heavy water labeling has been used in mice^71,72^, rats^69^, and humans^24^. Consumed ^2^H_2_O (dideuterated water, M + 2) rapidly equilibrates with body fluid, which in turn produces monodeuterated water (^2^H^1^HO) via exchanges of hydrogen (either ^1^H or ^2^H) between ^2^H_2_O (M + 2) and ^2^H^1^HO (M + 1). Due to the relatively slow turnover rate of body water (half-life: ~10 days)^24^, the water enrichment established following a dose is maintained for a relatively long time period, during which the ^2^H of water is incorporated into amino acids via transamination reactions, and the labeled amino acids are precursors for protein synthesis^24^. There is a “fixed” relation between water enrichment (M + 1) and amino acid enrichment (M + 1) because the number of ^2^H labeling sites for a specific amino acid is constant^26^. For example, there are theoretically 4 labeling sites of ^2^H from water in alanine^68^, but that value has been empirically found to be ~3.7 for mice, rats, and humans. Enrichment of the product (e.g., protein-bound alanine) is determined from muscle tissue sampled at the end of the experimental period divided by the precursor enrichment, as described above. Precursor amino acid enrichment may be determined directly. Alternatively, amino acid precursor enrichment may be determined indirectly from body water enrichment (reflected by any fluid in equilibrium with body water, such as saliva, plasma, urine, etc.) because of “fixed” relations between water enrichment and alanine enrichment.

#### Kinetic calculations

*FSR* is calculated using the tracer incorporation model discussed above, wherein the rate of incorporation of labeled precursor over time is divided by the enrichment of the precursor. Because water enrichment dictates the amino acid enrichment (E_AA_), the precursor enrichment can be expressed as the product of the sampled water enrichment (E_H2O_) and the number of deuterium labeling sites of the tracee amino acid: *FSR*(%/*t*) = [EAA/(E_H2O_ × # labeling site × *t*)] × 100^24^. It is assumed that *FSR* is constant (i.e., the function is linear) over the experimental time period, which may be problematic if sampling occurs over a prolonged interval. In the case of protocols spanning several days, it must be assumed that the various changes that occur in *FSR* within that time frame are averaged out by sampling at the beginning and end of the experimental period.

### “Virtual biopsy” technique—assessing muscle *FSR* using blood samples

The incorporation model is well suited to measuring muscle protein *FSR* but limited by the invasiveness of muscle biopsies. The “virtual biopsy” method has been developed to circumvent the requirement for biopsies to measure muscle protein *FSR* (Fig. 7c)^5^. Instead of measuring product enrichment directly from muscle biopsies, the method measures the enrichment of a circulating protein that is mostly released from muscle, such as creatine kinase muscle isoform (CK_M_, >90% from skeletal muscle) or carbonic anhydrase 3 isoform (CA-3, virtually specific to skeletal muscle)^6^. Using this approach, it was demonstrated in humans that there are strong correlations between muscle protein *FSR* and “virtual” *FSR* (*r* > 0.89 for CK_M_; *r* > 0.87 for CA-3)^5^ and between “virtual” *FSR* and myofibrillar proteins, including actin, myosin-2, and tropomyosin alpha-1 (for all, *r* > 0.66)^5^. Therefore, the “virtual biopsy” method may provide practical advantages in assessing muscle dynamics when muscle biopsies are not feasible despite its inability to differentiate among muscle tissues.

### Exploration of proteome kinetics—heavy water labeling combined with proteomics

Proteomics refers to the large-scale analysis of proteins. The abundance of hundreds or even thousands of individual proteins is determined in the same sample. Proteomics is conceptually linked to the description of the entire set of proteins produced in a biological system, which is called the proteome. Proteomics has become a widely used tool to examine the effects of nutritional or pharmacological treatments^73^. However, as explained in the example provided above regarding the limited kinetic information provided by measuring only the amount of water in the tank, proteomics is limited by the “static” nature of the data. Exploring dynamic changes in the proteome may represent an important advance in proteomic analysis. Measuring dynamic changes in the proteome for days to months with heavy water labeling in conjunction with proteomics can potentially solve the intrinsic limitation of proteomics^5,6^. With this kinetic approach, the dynamic changes of hundreds to thousands of proteins can be measured simultaneously. The proteome kinetics technique is categorized into four steps: (1) orally ingested ^2^H_2_O labels amino acids with deuterium, which are in turn incorporated into proteins; (2) labeled proteins are digested with trypsin to generate labeled peptides for analysis with high-resolution mass spectrometry, and individual proteins are identified from known specific sequences of peptides through database search; (3) turnover rates of individual proteins are determined based on the abundance and patterns of labeling of the peptides by the tracer incorporation technique; and (4) network analysis of *FSR* of individual proteins in the proteome can enable the identification of biologically meaningful groups of proteins (Fig. 7e). Note that this process relies on the single-precursor incorporation tracer model described above as applied to a variety of individual products.

### Multiple-pool, multiple-precursor model

**Fig. 8:**
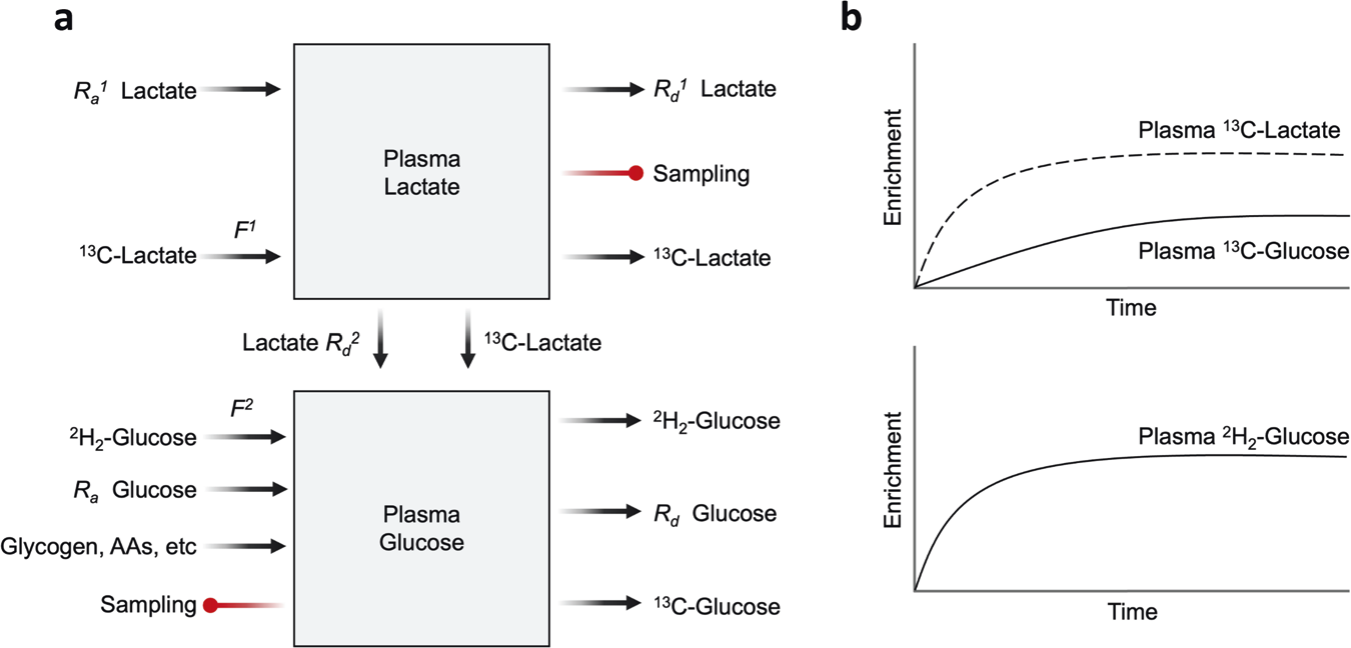
Multiple-pool, multiple-precursor model with glucose production from lactate as an example. **a** The ^13^C-lactate tracer is infused into plasma (pool 1). Lactate tracee enters pool 1 from various tissues, especially muscle (*R*_*a*_^1^). Lactate can leave pool 1 by various pathways, including glucose production (*R*_*d*_^2^). To determine the rate of glucose production from lactate (i.e., *R*_*d*_^2^), the fractional contribution of lactate to total glucose production must be calculated as the ratio of the plasma enrichment of ^13^C-glucose to that of ^13^C-lactate, multiplied by the total glucose production rate or *R*_*a*_ glucose, which is determined by a separate glucose tracer (e.g., ^2^H_2_-glucose). **b** ^13^C-glucose enrichment derived from ^13^C-lactate will never reach the plateau enrichment of plasma lactate because of the dilution of label incorporation by the incorporation of other unlabeled precursors (e.g., glycerol, amino acids, and glycogen). The difference in enrichment between ^13^C-glucose and ^13^C-lactate represents the contribution of other precursors, such as glycogen, various amino acids, and glycerol.

When a substrate such as glucose is produced from more than one precursor, the appropriate tracer model accounts for multiple-pool kinetics with multiple precursors for the production of a single product. For example, glucose is produced from multiple precursors, such as lactate, glycerol, pyruvate, and various amino acids^42,74–78^. Determination of the rate of oxidation from a specific substrate is another example of a multiple-pool, multiple-precursor model. In the latter example, CO_2_ is produced by oxidative phosphorylation in mitochondria from various substrates, including glucose, FFAs, amino acids, and multiple other compounds^79–81^. The multiple-pool, multiple-precursor model is distinct from the single-precursor model because the kinetics of substrates other than the tracee can influence the calculated rate of synthesis of the product. For example, consider the determination of the rate of glucose production from lactate using labeled lactate as the tracer in two different circumstances in which glucose production from lactate is equal. Calculation of the rate of glucose production from lactate by dividing the tracer enrichment in the product (glucose) by the precursor (lactate enrichment), as described above for a single precursor model, will provide only the fraction of glucose production from lactate in this case. The total rate of glucose production from lactate is not determined with only one tracer. The fraction of glucose production from lactate must be multiplied by the total rate of glucose production to calculate the rate of glucose production from lactate. The rate of total glucose production must be determined independently by tracer dilution of a glucose tracer, such as 6,6 ^2^H_2_-glucose. The reason that a multiple-precursor model is required to calculate the rate of glucose production from lactate is that variable rates of glucose production from other precursors, such as glycerol, amino acids, and glycogen, will affect the total rate of production of glucose and thus the fraction of glucose production from lactate. In this example, the measured fraction of glucose derived from lactate (i.e., product enrichment divided by precursor enrichment) will be reduced by an elevated total rate of glucose production, even if the absolute rate of glucose production from lactate remains constant. Multiplication of the fraction of glucose derived from lactate by the total rate of glucose production is necessary to obtain meaningful data. Thus, in contrast to the single-precursor model, the labeling of the product in a multiple-precursor model is not necessarily a reflection of the rate of production from the labeled precursor. Rather, it is necessary to consider the total rate of production of the product, which must be determined by the tracer dilution method independently of the incorporation of the tracer of the precursor of interest. In this section, the impact of possible isotopic exchange in the TCA cycle will not be considered, as we have focused on the structure of the tracer model. The principle of the multiple-pool, multiple-precursor model is shown schematically in Fig. 8.

### Metabolic flux analysis

**Fig. 9:**
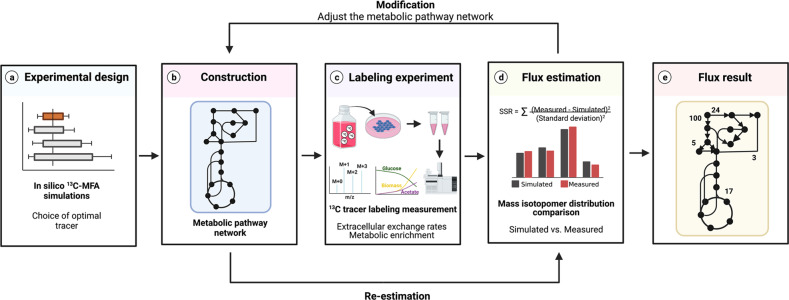
Overview of procedures for metabolic flux analysis (MFA). MFA is performed as follows: **a** Selection of isotopic labeling experiments; **b** metabolic network model construction; **c** stable isotope tracer labeling experiments and measurement of isotopic labeling patterns of metabolites derived from the tracer substrate; **d** estimation of intracellular metabolic fluxes using statistical analysis; and assessment of flux values by using the goodness of fit and confidence intervals. **e** Results of metabolic flux rates of the pathway. RSS, residual sum of squares.

The quantification of intracellular metabolic flux (e.g., glycolysis, the pentose phosphate pathway, the Krebs cycle, etc.) has long been of interest because of the important roles these pathways play in maintaining metabolic homeostasis. However, it is generally not possible to quantify intracellular metabolic flux rates with the traditional application of tracer methodology. The problem is that most components of metabolic pathways can be produced from multiple precursors, in which case the multiple-pool, multiple-precursor model applies. This model requires the measurement of both the rate of incorporation of labeled precursor into the product and the total rate of intracellular production of the product. The latter value is generally not accessible in an in vivo experiment designed to assess multiple intermediates in a metabolic pathway. Metabolic flux analysis (MFA) with the use of stable isotope tracers is an approach to approximate the flux rates of components of metabolic pathways without having all the data necessary to directly measure the rates^82–84^. MFA estimates flux rates through several steps, as represented schematically in Fig. 9. The first step is the selection of the optimal tracer and construction of the stoichiometric matrix model. The selection of optimal tracers is determined via in silico simulations (i.e., computer-based simulation)^85^, and databases (e.g., the Kyoto Encyclopedia of Genes and Genomes) can be used to construct the stoichiometric matrix model. The next step is to perform tracer labeling experiments and measure isotopic labeling patterns and external rates (i.e., substrate uptake and release rates). Since isotopic labeling pattern data provide only relative information, external rates are required to quantify intracellular metabolic flux rates. The third step is flux estimation, which can be accomplished by several software tools, including INCA and Metran, which use the elementary metabolite unit (EMU)^86^ framework for the simulation of isotopic labeling and flux. The estimated flux results are modified to minimize the residual sum of squares (RSS) between simulated and observed results by adjusting the metabolic network model^87^. A lower RSS indicates better agreement between the simulated and observed results. Since the minimized RSS is a random variable with a *χ*^2^ distribution, the confidence intervals of the RSS can be calculated to verify the flux result. If the flux result is within the statistically acceptable range (e.g., 0.05 for the 95% confidence intervals), it is considered to represent the absolute rates of intracellular metabolic flux. While MFA can provide quantitative flux rates, it should be recognized that the method has been developed to overcome the fact that the requirements of the multiple-precursor, multiple-product model cannot be satisfied. While the precision of results from MFA can be assessed statistically, empirical validation of its accuracy is usually not possible.

## Summary and conclusions

Living organisms are in a constant state of metabolic turnover. Traditional “omics” and molecular tools provide static, snapshot information, collectively termed “statomics”. Stable isotope tracer methodology, on the other hand, provides dynamic data that can quantify metabolic flux rates. Tracer methods are all predicated on two basic model structures, i.e., tracer dilution and tracer incorporation. The basic model structures can be further divided according to the number of pools (single pool or multiple pool) and/or the number of precursors (i.e., single or multiple precursor). Use of tracer dilution alone or together with tracer incorporation can enable the assessment of in vivo and in vitro dynamics of various biological compounds. The tracer dilution model is also applicable to other usages, including direct assessment of functional muscle mass based on the dilution of consumed D_3_-creatine by the existing unlabeled creatine pool. A key element of tracer methodology is that the tracer labeling of a product, regardless of the model structure, provides fractional information but does not provide actual rates without knowledge of pool size in the case of a single precursor or total production rate in the case of the multiple-precursor model. The use of heavy water labeling has increased over the past decades due to the minimally invasive nature of the method and the diverse array of potential metabolic precursors and products that can become labeled in vivo. However, when using heavy water labeling, the basic principles of the model structure still apply. On the other hand, traditional tracer methodology generally will not apply to the measurement of multiple flux rates in intracellular metabolic pathways. Metabolic flux analysis (MFA) enables the approximation of flux rates that cannot be directly quantified by traditional tracer methodology.
